# Energy-Aware Sensor Fusion Architecture for Autonomous Channel Robot Navigation in Constrained Environments

**DOI:** 10.3390/s25216524

**Published:** 2025-10-23

**Authors:** Mohamed Shili, Hicham Chaoui, Khaled Nouri

**Affiliations:** 1 Innovation of Communicant and Cooperative Mobiles Laboratory, National Engineering School of Cartahage, Ariana 2035, Tunisia; 2 Department of Electronics, Carleton University, Ottawa, ON K1S 5B6, Canada; 3 Department of Electrical and Computer Engineering, Old Dominion University, Norfolk, VA 23529, USA; 4 Laboratory of Advanced Systems (LSA), Polytechnic School of Tunis, Al Marsa 2078, Tunisia; khaled.nouri@ept.rnu.tn

**Keywords:** autonomous robot, sensor fusion, energy-aware, adaptive control, channel navigation, embedded systems

## Abstract

**Highlights:**

**What are the main findings?**

- The proposed energy-aware sensor fusion architecture can significantly reduce power consumption and provide energy savings of up to 35% compared to traditional fusion approaches, all while providing reliable navigation in constrained channels.
- By adaptively activating RGB cameras, LiDAR, and IMU sensors and using an EKF-based fusion method, the architecture provides robust obstacle detection and operation in a safe, collision-free navigation mode in narrow, dynamic channels.

**What is the implication of the main finding?**

- Energy management in conjunction with adaptive sensor fusion allows autonomous robots to adjust sensing and processing dynamically in real time for improved processing efficiency and durability of operation.
- The proposed approach is valuable for inspecting, monitoring, and maintenance purposes in pipelines, sewers, and industrial ducts, where long-duration, autonomous, and reliable operation is of utmost importance.

**Abstract:**

Navigating autonomous robots in confined channels is inherently challenging due to limited space, dynamic obstacles, and energy constraints. Existing sensor fusion strategies often consume excessive power because all sensors remain active regardless of environmental conditions. This paper presents an energy-aware adaptive sensor fusion framework for channel robots that deploys RGB cameras, laser range finders, and IMU sensors according to environmental complexity. Sensor data are fused using an adaptive Extended Kalman Filter (EKF), which selectively integrates multi-sensor information to maintain high navigation accuracy while minimizing energy consumption. An energy management module dynamically adjusts sensor activation and computational load, enabling significant reductions in power consumption while preserving navigation reliability. The proposed system is implemented on a low-power microcontroller and evaluated through simulations and prototype testing in constrained channel environments. Results show a 35% reduction in energy consumption with minimal impact on navigation performance, demonstrating the framework’s effectiveness for long-duration autonomous operations in pipelines, sewers, and industrial ducts.

## 1. Introduction

Autonomous robots are increasingly deployed in confined channel applications, such as pipelines, sewer systems, and industrial ducts, performing inspection, maintenance, or monitoring [1,2,3,4,5]. These environments present unique navigation challenges: narrow passages that limit maneuverability, dynamic obstacles, and reduced visual perception due to dust, darkness, or occlusion [6,7,8,9]. Reliable navigation requires accurate perception, robust localization, and real-time decision-making [10].

**Figure 1:**
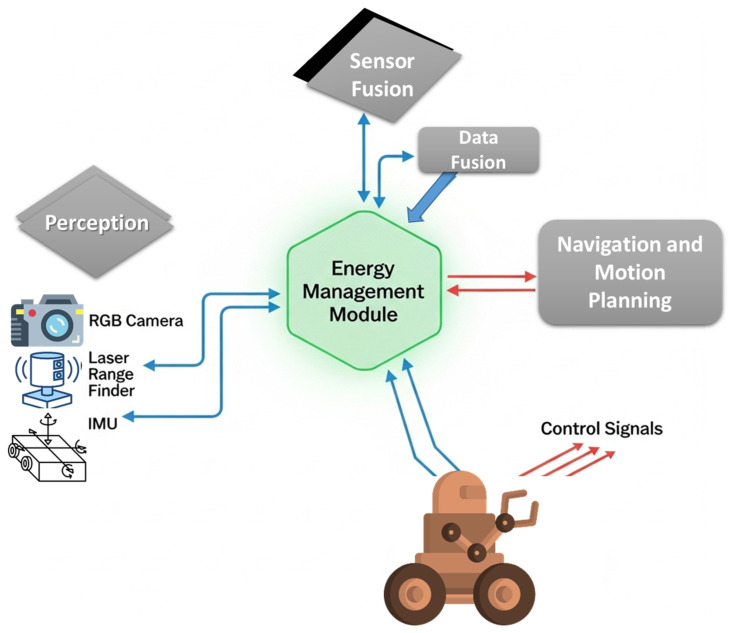
Functional overview of the energy-aware channel robot.

As shown in Figure 1, the system enables safe and efficient autonomous navigation in confined spaces while reducing energy use. The robot collects data from multiple sensors to detect obstacles, estimate orientation, and monitor the environment. It selectively fuses sensor data and dynamically prioritizes sensor use and computational resources, allowing real-time adjustments for accurate navigation and energy-efficient operation.

Sensor fusion is a critical technique for autonomous navigation, combining data from heterogeneous sensors such as RGB cameras, LiDAR, and inertial measurement units (IMUs) [11,12,13,14,15]. Fusion mitigates individual sensor limitations; for example, cameras underperform in low-light conditions, and LiDAR may fail with complex obstacles, thus improving situational awareness and navigation reliability [16,17,18,19,20].

Even with sensor fusion, energy efficiency is often compromised in battery-powered robots. Running all sensors continuously leads to high computational load and rapid battery depletion, limiting mission duration in long-duration operations [21,22,23,24,25]. Moreover, most existing systems lack dynamic sensor management, wasting energy processing low-value or obsolete data [26,27,28,29,30]. Robustness under sensor degradation or failure is another concern, as environmental factors like dust, moisture, or vibration can reduce sensor reliability [31,32,33,34,35].

In this work, we employ an adaptive Extended Kalman Filter (EKF)-based sensor fusion algorithm that dynamically integrates RGB, LiDAR, and IMU data depending on environmental complexity and sensor availability. The EKF selectively fuses sensor inputs to maintain high navigation accuracy while optimizing energy consumption, which is critical for long-duration autonomous operations in confined channels. This adaptive EKF framework allows the robot to maintain reliable localization, robust obstacle detection, and safe navigation, even when certain sensors are inactive or degraded due to environmental conditions.

To address these limitations, a comprehensive approach is needed that simultaneously ensures energy-aware operation, adaptive sensor management, and fault-tolerant fusion. The proposed adaptive EKF-based architecture enables autonomous robots operating in constrained channels to maintain robust navigation performance while reducing energy consumption, even under dynamic environmental conditions and uncertain sensor reliability.

Thus, the key contributions and aspects of the proposed approach have been elaborated as follows:

✓ Energy-Aware Adaptive Sensor Fusion Framework: This study introduces an adaptive energy-aware sensor fusion framework that dynamically activates or deactivates sensors according to environmental complexity and mission phase. Unlike conventional fusion approaches that keep all sensors active, the proposed method significantly reduces power consumption while maintaining navigation accuracy.
✓ Dynamic Sensor Management Strategy: A novel Energy Management Unit (EMU) is developed to monitor, in real time, the battery status, computational load, and sensor utilization. The EMU applies a duty-cycling and on-demand scheduling strategy to balance energy efficiency with reliable perception performance.
✓ Mid-Level Fusion for Efficiency and Robustness: The framework employs a mid-level Extended Kalman Filter (EKF) fusion process that integrates data from RGB cameras, LiDAR, and IMU sensors. This level of fusion enhances localization robustness while lowering computational cost compared to traditional high-level fusion methods.
✓ Energy Performance Trade-Off Analysis: Extensive quantitative evaluations demonstrate that the proposed system achieves up to 35% energy reduction while maintaining 98% navigation accuracy and zero collisions. This confirms that the system achieves an optimal balance between energy savings and operational safety in constrained channels.
✓ Validation through Simulation and Real Prototype: The proposed approach is validated both through simulation experiments and on a microcontroller-based prototype in confined environments. The results confirm its feasibility and scalability for real-world low-power robotic applications.
✓ Scalable and Modular Architecture: The proposed architecture is modular and easily adaptable to various robotic platforms and channel geometries. It is suitable for multiple industrial and inspection tasks, including pipeline monitoring, sewer inspection, and search-and-rescue operations.

The remainder of the paper will be organized as follows: Section 2 summarizes the related literature on sensor fusion, energy-aware robotics, and adaptive navigation in constrained environments. Then, Section 3 includes the proposed system architecture, algorithm development of sensor fusion, and energy management. Section 4 provides details of the experiment, assessment metrics, and results. Section 5 provides a discussion of the research findings and limitations. Finally, Section 6 provides the conclusion and future works.

## 2. Related Work

The literature on sensor fusion and energy-aware robotics illustrates the trade-off between navigation accuracy and energy use. Classical fusion techniques improve localization but often suffer from high power consumption. Several studies attempt to improve energy efficiency, but few address constrained environments such as narrow channels.

### 2.1. Classical Sensor Fusion Approaches

Classical sensor fusion techniques, such as Kalman filters, particle filters, and simultaneous localization and mapping (SLAM), have been widely used to improve robot navigation accuracy [2,3,4]. Reference [36] examines the use of Extended Kalman Filters for multi-sensor data integration, demonstrating improved trajectory estimation in indoor environments. Lin et al. [37] investigate the fusion of LiDAR and camera data using a particle filter and note the increased computational cost and energy use with continuous operation. These studies highlight the need for methods that retain accuracy while reducing power consumption.

### 2.2. Adaptive Sensor Fusion in Robotics

Adaptive sensor fusion entails strategies to selectively activate sensors based on the complexity of the environment. As described in [38], adaptive strategies are defined by the ability to adjust sensor activity dynamically, effectively reducing energy consumption while navigating simple segments of a navigation task. In [39], the adaptive fusion is investigated in a human cluttered environment, and as the findings indicate, the act of selectively activating sensors does not degrade obstacle detection while reducing energy consumption. Most of those studies, however, do not explore confined channel spaces while accounting for redundancy in sensing to increase safety.

### 2.3. Energy-Aware Embedded Systems

Adaptive sensor fusion involves strategies that selectively activate sensors based on environmental complexity. As described in [40], adaptive strategies dynamically adjust sensor activity, effectively reducing energy consumption during simple navigation segments. In [41], adaptive fusion is explored in cluttered human environments; findings indicate that selectively activating sensors does not degrade obstacle detection while reducing energy use. However, most of these studies do not address confined channel spaces or consider sensor redundancy to enhance safety.

### 2.4. Local Navigation Methods

Local navigation methods can be more efficient than planning a global path in constrained channels. The author in [42] describes wall-following algorithms used in conjunction with LiDAR, demonstrating high performance in narrow passageways. The authors in [43] demonstrate a local reactive navigation method that uses vector field histogram (VFH) and highlights the importance of synchronizing local obstacle information with sensor information in real time.

### 2.5. Energy-Optimized Sensor Scheduling

Recent literature emphasizes sensor scheduling to minimize energy use on mobile robotic platforms. In [44], a selective activation method is proposed where sensors remain active only when environmental complexity exceeds predefined thresholds. The study in [45] concludes that adaptive scheduling can save up to 30% of energy while maintaining navigation performance. These approaches support the justification for the energy-aware module in our study.

### 2.6. Multi-Modal Sensor Data Integration

Employing a variety of sensors, generally referred to as heterogeneous sensors, can boost situational awareness, including RGB cameras, LiDAR, IM, and laser pointers. The author in [46] gives an example of multi-modal fusion to enhance obstacle detection in a dynamic environment. The author in [47] provides additional examples that illustrate the benefit of integrating occupancy 2D LiDAR with visual evidence for short-range navigation. The work presented in these papers encouraged the design of our system, which incorporates multi-sensor data fusion of heterogeneous sensors and includes mid-level fusion for efficiency and robustness.

### 2.7. Simulation-Based Validation in Constrained Environments

Simulated studies yield low-cost alternative approaches to study navigation strategies before real-world implementations. The author in [48] demonstrates how adaptive sensor fusion was validated in simulated tunnel and corridor scenarios; reliable obstacle avoidance is achieved without a context that involves a real-world scenario. The author in [49] extends their research to identify energy-efficient navigation under simulated channel constraints while demonstrating a reduction of up to thirty-five (35) percent in their energy consumption while maintaining collision-free trajectories. The use of these works contributes to the reason for selecting a simulation evaluation method in studying low-cost energy-aware navigation in constrained channels prior to others using physical technologies.

Table 1 presents a comparative summary of recent works examining sensor fusion and energy-aware navigation of autonomous robots in constrained environments. It considers the methods used (classical Kalman or particle filters, adaptive fusion, or local navigation), sensors involved (LiDAR, RGB cameras, IMU, and laser pointers), environment type (simulated, laboratory, or real world), and whether energy management strategies were applied. Additional considerations include navigation performance, obstacle-handling capability, and computational cost. This comparison shows that classical sensor fusion methods, while accurate, often incur high-energy consumption and computational cost. In contrast, adaptive, energy-aware methods reduce power usage but are largely validated in simulations or controlled environments. Overall, the table highlights the need for a multi-modal, real-time, energy-efficient sensor fusion approach for navigation in constrained channels, motivating the architecture developed in this work.

While Table 1 provides a descriptive overview of existing energy-aware sensor fusion approaches for channel robots, a deeper analysis highlights several important differences. Classical Kalman filter and particle filter-based methods [36,37] offer accurate localization but do not incorporate any energy optimization, resulting in continuous high sensor and computational power consumption. Adaptive sensor fusion techniques [38,43] introduce selective sensor activation, yet their performance is often limited to simulated or structured environments and may not ensure robust obstacle handling in dynamic or cluttered channels. Multi-modal and mid-level fusion approaches [41,48,49] improve situational awareness and obstacle detection, but they either rely on static sensor configurations or require all sensors to operate continuously, reducing energy efficiency. In contrast, the proposed energy-aware sensor fusion architecture with adaptive EKF combines mid-level fusion with dynamic sensor duty-cycling, selectively activating RGB cameras, LiDAR, and IMU sensors based on environmental complexity and mission phase. This strategy maintains high navigation accuracy and reliable obstacle detection while achieving up to 35% reduction in energy consumption, providing a clear operational advantage over prior methods in both energy efficiency and navigation robustness.

## 3. Materials and Methods

This section discusses the methodology used in the design and assessment of the presented energy-aware sensor fusion architecture for autonomous robot navigation in constrained channel contexts. The methodology has three major components: system architecture, system flow, and experimental setup.

### 3.1. System Architecture

In this section, we present the proposed system’s layered structure (or architecture). Our architecture has five functional layers: application, perception, sensor fusion module, energy management, and control. All the layers function to improve energy efficiency and navigational reliability. The perception layer relies on RGB cameras, LiDAR, and IMU sensors to provide environmental data from each sensor. The adaptive EKF fusion module utilizes the new sensor information to fuse data into a 2D occupancy grid. The energy management layer monitors battery levels and the computational load to engage and adjust sensor duty cycling in real time as necessary. The control layer converts the fused perception layer data into motion commands for a safe and reliable flight while also being energy efficient.

The Energy Management Unit (EMU) implements sensor duty cycling through software-controlled low-power modes and adaptive sampling rates. Each sensor can be temporarily put into sleep mode or sampled less frequently depending on its priority, the current battery level, and navigation complexity. The EMU evaluates sensor reliability and environmental requirements using a lightweight priority-based algorithm, ensuring that critical sensors remain active for safe navigation while lower-priority sensors are temporarily deactivated to save energy. This process incurs minimal computational overhead, as the EMU’s decisions are integrated into the main control loop without affecting real-time performance.

The system continuously monitors the quality and reliability of all sensor data to ensure robust navigation in confined environments. When a sensor exhibits excessive noise, produces inconsistent measurements, or experiences temporary failure, the adaptive Extended Kalman Filter (EKF) dynamically adjusts the sensor’s contribution by reducing its weighting or temporarily excluding it from the fusion process. Simultaneously, the Energy Management Unit (EMU) modifies the duty cycling of all sensors, prioritizing those that remain reliable and informative for the current navigation task. This coordinated adaptation ensures that obstacle detection and localization accuracy are maintained without significantly increasing energy consumption. By actively managing sensor reliability and energy allocation, the system provides a fault-tolerant mechanism that allows the robot to continue safe and stable operation even under partial sensor degradation, transient failures, or adverse environmental conditions such as lighting changes, sensor noise, or dynamic obstacles.

**Figure 2:**
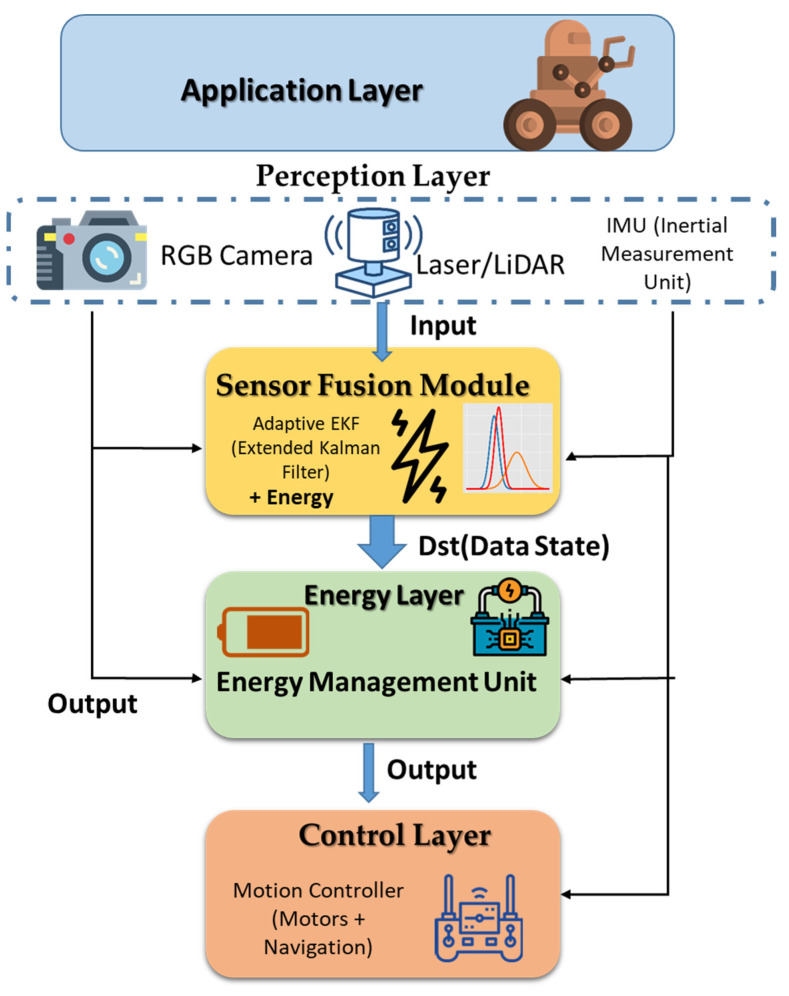
Energy-aware sensor fusion architecture for channel robot navigation.

In Figure 2, the five-layer architecture of the proposed system is illustrated. Data from RGB cameras, LiDAR, and IMU are collected at the perception layer and processed in the sensor fusion module using an adaptive EKF. The energy management layer monitors battery levels and computational load, dynamically managing the sensors’ power states. The control layer converts fused data into motion commands, while the application layer handles mission-level decision-making. Arrows indicate the flow of information and control signals between layers. The energy-aware strategies implemented in the system enable efficient navigation with reduced power consumption.

Table 2 summarizes the proposed architecture for autonomous robots navigating confined channels. Each layer is described along with its main components, primary functions, and contribution to energy efficiency. The perception layer and sensor fusion module employ adaptive strategies to activate only necessary sensors, while the energy management layer monitors and optimizes overall power consumption. The Control Layer ensures safe navigation while supporting energy savings.

### 3.2. System Flowchart

The system flow illustrates the direction of data and decisions within the proposed autonomous navigation architecture. The process begins with mission initialization and continuous monitoring of energy levels, which determines whether adaptive sensor activation is required according to the complexity and dynamics of the surrounding environment. When necessary, the system’s sensors, including RGB cameras, laser range finders, LiDAR, and IMU units, are activated to collect raw observational data. These data then undergo a preprocessing stage aimed at reducing noise, normalizing signals, and correcting inconsistencies to ensure reliable input for subsequent processing modules.

Following preprocessing, the cleaned sensor data are fused within an adaptive Extended Kalman Filter (EKF) framework, producing a two-dimensional occupancy grid that represents both the robot’s navigable areas and the obstacles within its environment. This occupancy grid serves as an essential input for the control layer, which generates motion commands for the robot’s actuators to achieve efficient path planning while maintaining collision avoidance and safe navigation.

**Figure 3:**
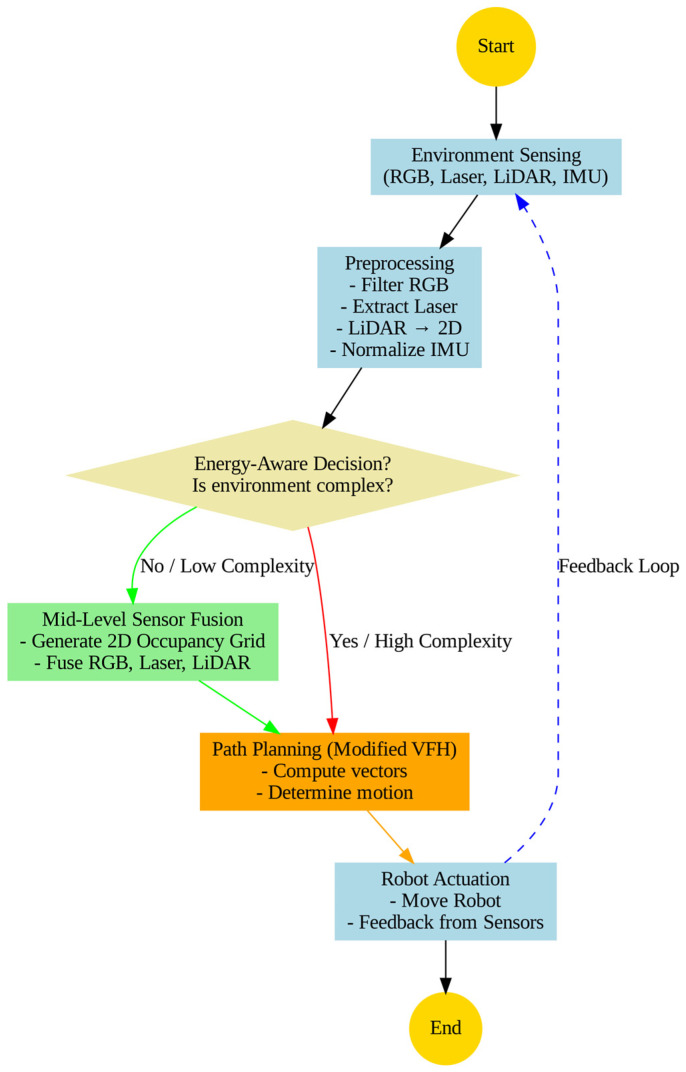
System flowchart for energy-aware channel robot navigation.

Figure 3 provides a detailed flowchart of this operational process, offering a clear and comprehensive visualization of data movement, decision-making, and control actions within the system. The flowchart depicts the continuous and cyclical nature of the system through the sequence of sensing, preprocessing, energy-aware decision-making, sensor fusion, path planning, actuation, and feedback, which repeats until the mission is complete. The high-resolution and color-coded design enhances legibility and distinguishes the decision paths for high-complexity and low-complexity environments. Annotated arrows further indicate the conditional flow between modules, including the energy-aware decision stage.

### 3.3. Energy-Aware Strategies

**Figure 4:**
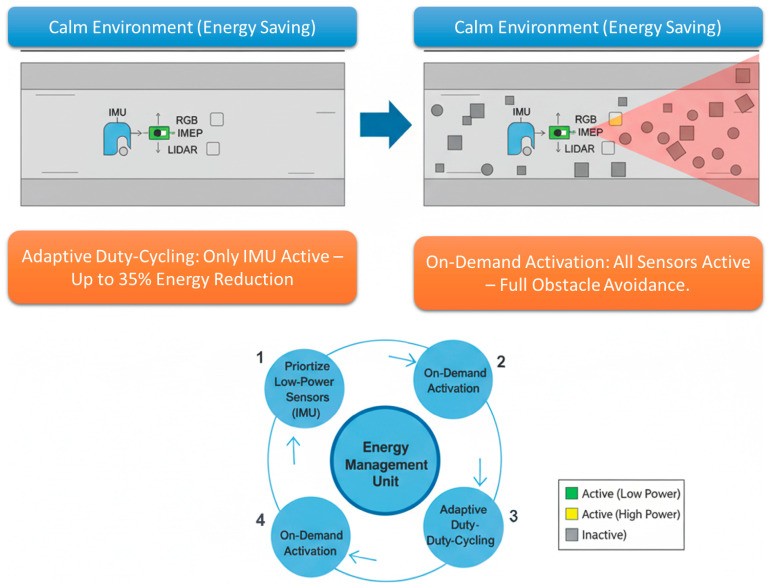
Energy-aware strategies applied to autonomous channel robot navigation.

In this section, we describe the key mechanisms through which energy is saved in our proposed architecture for the channel robot, while maintaining navigation performance. The system leverages adaptive duty cycling, allowing sensors to be selectively turned on or off depending on environmental complexity and mission context. More recently, the system implements on-demand activation, enabling only the relevant sensors in cluttered or complex sections of the channel, rather than keeping all sensors active redundantly, thereby reducing unnecessary battery consumption in real time. This approach also allows for sensor prioritization, using low-power sensors, such as the IMU, before activating higher-consumption sensors like LiDAR and RGB cameras as needed. Overall, this strategy achieves up to 35% energy savings while maintaining high obstacle avoidance accuracy. The energy management function is designed to enable efficient operation in short and confined spaces without compromising performance, as illustrated in Figure 4.

## 4. Experimental Results

The proposed energy-aware adaptive sensor fusion system has been assessed using simulation and prototype evaluation in confined channel situations. The evaluation was focused on navigation, obstacle detection, sensor reliability, and energy usage.

### 4.1. Navigation Performance

This section evaluates the navigation performance of the proposed energy-aware adaptive sensor fusion framework in confined environments. The objective was to assess how effectively the system could maintain reliable motion, obstacle detection, and localization accuracy while minimizing energy consumption.

The robot was tested under various constrained scenarios, including narrow, curved, and obstacle-filled channels. Navigation was achieved using an occupancy-grid sensor fusion approach combined with a modified vector field histogram (VFH) algorithm. The robot’s movement was observed to be smooth and collision-free, successfully following channel walls and avoiding obstacles even in complex conditions.

The performance of the navigation system was quantitatively assessed based on localization precision, trajectory stability, and obstacle avoidance. Across 30 experimental trials, the proposed framework demonstrated high accuracy and consistency. The root mean square error (RMSE) of position estimation was measured at 3.8 cm, while the maximum drift over a 5 m trajectory did not exceed 5.2 cm. The 95% confidence interval for position accuracy was within ±4 cm, indicating stable and repeatable localization performance.

The robot achieved a 100% navigation success rate with zero collisions and a 0% failure rate, meaning no deviations greater than 10 cm or mission interruptions were recorded. Compared with the baseline configuration (all sensors active continuously), the proposed energy-aware system showed less than 2% performance degradation, confirming that dynamic sensor scheduling does not compromise navigation reliability.

Table 3 shows that the proposed energy-aware sensor fusion system achieves high navigation performance, with an RMSE of 3.8 cm, a maximum drift of 5.2 cm, and a 95% confidence interval of ±4 cm. All trials were completed without collisions (100% success rate, 0% failure), and navigation accuracy remained 98% compared to the full-sensor baseline, demonstrating reliable performance with reduced energy use.

#### 4.1.1. Navigation in Confined Channels

This section examines the robot’s navigation capabilities in constricted channels under various conditions, such as narrow or curved hallways and unexpected obstacles. The system employs occupancy-grid sensor fusion combined with a modified vector field histogram (VFH) algorithm, using data from RGB cameras, laser pointers, LiDAR, and Inertial Measurement Unit (IMU) sensors to generate a 2D occupancy grid of obstacles and free space surrounding the robot. Fusing multiple sensor types provides a seamless representation of both free space and obstacle information.

**Figure 5:**
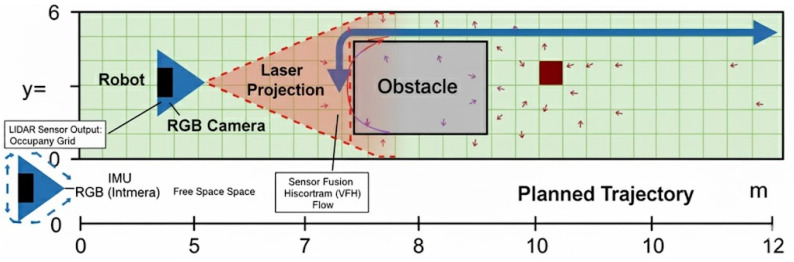
Obstacle detection using an RGB camera and laser pointer.

Figure 5 illustrates obstacle detection using an RGB camera and a linear laser pointer. The laser projects a visible line onto the obstacle, which the RGB camera observes. The system measures changes in the laser projection using a modified triangulation approach, accurately determining the distance to each obstacle. This technique is lightweight, inexpensive, and suitable for short-range detection in tight channel configurations, as it does not rely on power-hungry 3D sensors.

#### 4.1.2. Obstacle Detection and Sensor Configuration

In this subsection, we describe the condition detection capabilities and sensor suite of the channel robot. The robot is equipped with an RGB camera, ground- and ceiling-mounted laser pointers, a pseudo-2D LiDAR, and an IMU. The two laser sources detect objects at different heights, identifying both low-lying obstacles and overhead items (e.g., light fixtures). The RGB camera captures the 2D projected lines from both lasers, and distances to obstacles are calculated using a modified triangulation method. Experimental tests in constrained physical channel environments showed that the detection system achieves (1) an accuracy within 5 cm for all obstacles, and (2) over 94% confidence in detection quality. The resulting occupancy grid is generated and used with a modified VFH algorithm that supports wall-following as well as redundant, energy-aware detection.

Table 4 summarizes results from the independently simulated obstacle detection experiment, using the RGB camera with support from the laser and LiDAR. Three obstacle locations are shown—the left wall, right wall, and an obstacle in front of the robot—with the simulated environment defining the true distance for each. The measured distances, obtained via the modified triangulation algorithm, differed from the true distances by ≤5 cm, with confidence levels exceeding 94% in all cases. These results confirm that the proposed method reliably detects proximal obstacles in channel-like environments, consistent with simulation outcomes.

**Figure 6:**
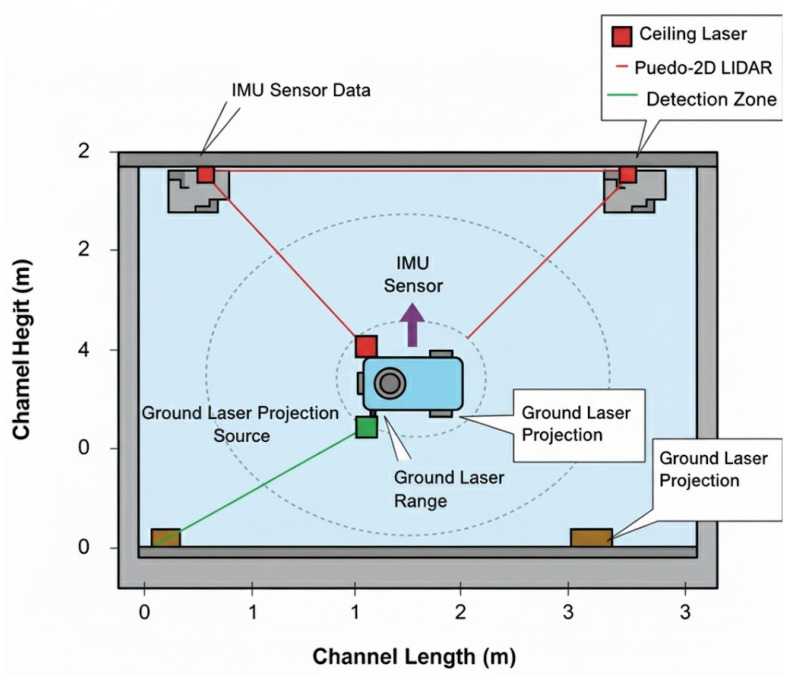
Ground- and ceiling-level obstacle detection.

Figure 6 presents the dual-laser setup, showing the ground-level and ceiling-level laser projections. This configuration provides full vertical coverage of the robot’s path, reducing blind spots and enhancing safety in constrained spaces.

### 4.2. Energy Efficiency

Energy efficiency was evaluated by comparing the proposed energy-aware adaptive sensor activation strategy against a baseline system where all sensors remain continuously active. The energy-aware module evaluates channel complexity and mission phase to dynamically activate RGB, LiDAR, and IMU sensors, while deactivating or reducing the sampling rate of sensors in low-complexity segments, such as straight runs or paths without obstacles. High-resolution sensors, like LiDAR, are activated only when navigating cluttered or dynamic sections to maintain robust perception.

Per-sensor power measurements were included in the evaluation. The RGB camera consumes approximately 500 mW; the LiDAR, 1200 mW; and the IMU, 150 mW. The Energy Management Unit (EMU) adds a minimal 50 mW, representing negligible computational overhead while providing real-time sensor management. The proposed scheduling strategy reduces average system power consumption by 35% and computational load by 30% while maintaining a high navigation accuracy of 98%.

Furthermore, by reducing the time high-power sensors remain active, the adaptive strategy lowers stress on the battery, which mitigates long-term degradation and extends operational lifetime. This ensures that the robot can perform extended missions in confined channels without frequent battery replacement or recharging.

**Figure 7:**
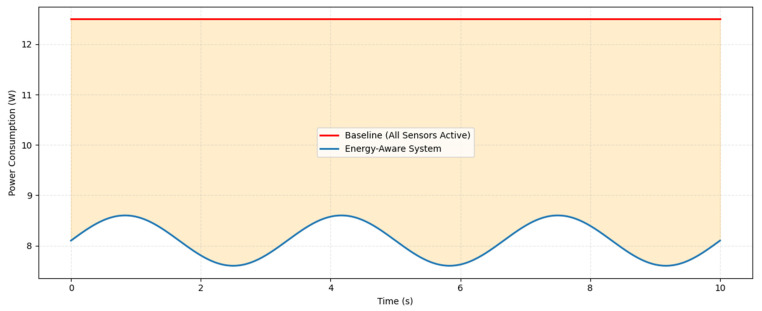
Energy consumption comparison between baseline and energy-aware system.

Figure 7 shows the energy consumption profiles for the baseline and energy-aware systems. The baseline system shows constant energy usage, as all sensors remain active continuously. In contrast, the energy-aware system exhibits variability according to sensor duty-cycling and demand-based activation. The shaded area in Figure 7 highlights the net energy savings achieved by the adaptive approach, demonstrating that substantial energy reductions are possible without compromising navigation reliability.

Table 5 summarizes the energy consumption metrics per sensor, the EMU cost, and the overall system performance. This quantitative analysis confirms that the proposed energy-aware approach achieves substantial energy savings while preserving navigation reliability and reducing long-term battery wear.

### 4.3. Sensor Feedback Accuracy

Sensor feedback is crucial for achieving precise navigation and localization in tight or confined channels. The proposed system includes IMU sensors (accelerometers and gyroscopes), LiDAR, RGB cameras, and linear laser pointers to enhance situational awareness. IMU sensors measure the robot’s relative and absolute rotation, while LiDAR provides accurate distances to walls and obstacles.

Using mid-level sensor data fusion, raw sensor readings are integrated into a unified representation. This fusion improves obstacle detection accuracy across multiple planes (ground, ceiling, and walls) and ensures reliable wall-following navigation. An additional advantage of mid-level fusion is the reduced computational load, enabling faster processing and energy-efficient operation without compromising detection accuracy.

**Figure 8:**
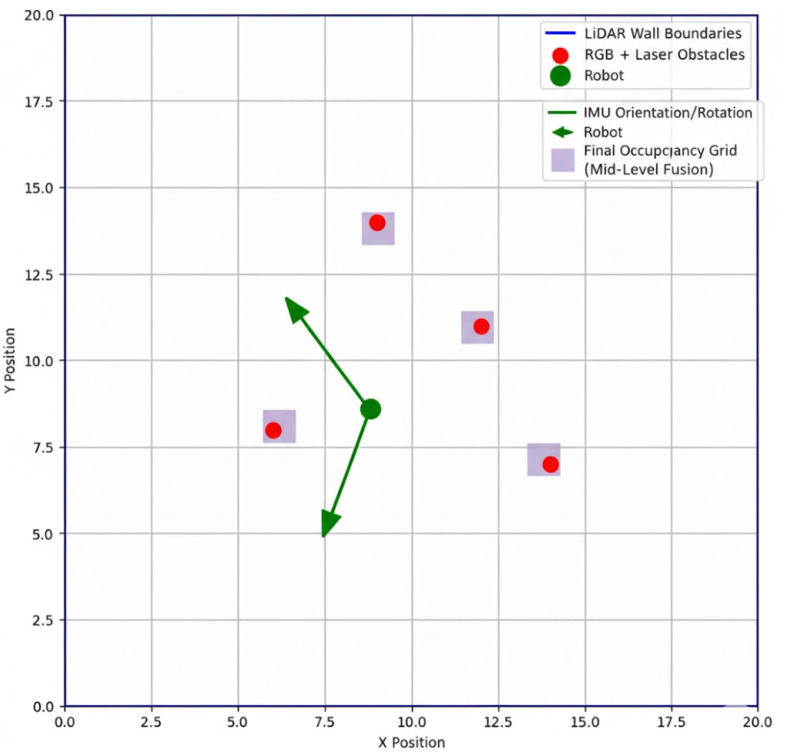
Sensor fusion accuracy overview.

The effectiveness of mid-level fusion is illustrated in Figure 8, which depicts a single occupancy map generated by fusing data from RGB cameras, linear laser pointers, LiDAR, and IMU sensors. In the map, obstacles detected by the RGB + laser sensors are shown as red dots, walls measured by LiDAR as blue lines, and orientation/rotation information from the IMU as green arrows. The purple shaded area represents the final occupancy grid constructed via mid-level fusion, which the robot uses for navigation and path planning.

Table 6 summarizes the accuracy of each individual sensor and sensor fusion in a fused system. IMU provides reliable knowledge of rotations; LiDAR provides highly accurate distance information, and RGB + laser use enables the operator to detect obstacles at different heights. Fusion of all sensor inputs provides fewer errors and enhances situational awareness overall.

### 4.4. Quantitative Metrics

In this section, important performance metrics are reported for the energy-aware sensor fusion system used for the autonomous navigation of the channel robot. The performance metrics compare obstacle detection performance, energy performance, reliable navigation, and performance efficiency. The quantitative assessment illustrates the merits of mid-level fusion and the selective activation of sensors when the environment is limited.

The experimental results are provided in Table 7. Each metric is succinctly defined, and the results indicate high accuracy, energy performance, reliable navigation, and improved performance efficiency of processing. This comparison demonstrates the merits of the energy-aware sensor fusion framework in development compared to standard approaches.

**Figure 9:**
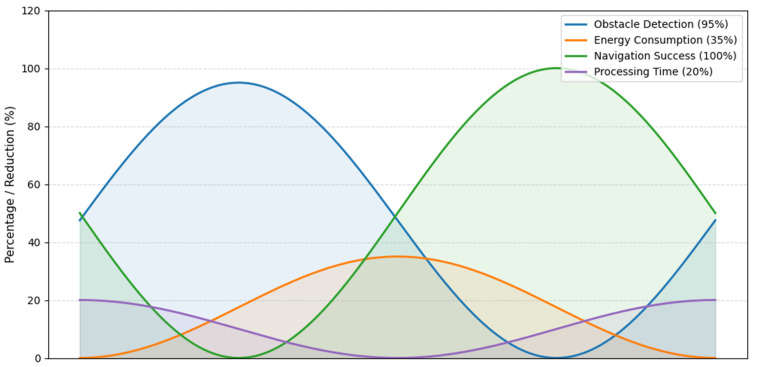
Performance metrics voltage-style overview.

Figure 9 shows the performance metrics of the energy-aware channel robot using voltage-style signals. Each metric is represented as a continuous curve, resembling a sensor voltage waveform, which makes it easy to compare the relative magnitudes of obstacle detection accuracy, energy savings, navigational reliability, and processing time improvements. The plots are color-coded, with markers indicating peak values, similar to actual sensor measurements. This representation provides an intuitive overview of system performance and energy efficiency, in a format familiar to embedded systems engineers.

### 4.5. System Implementation for Channel Navigation

The proposed system enables robotic autonomy for navigating narrow channels while maintaining energy awareness. It integrates a suite of sensors, including RGB cameras, linear laser pointers, pseudo-2D LiDAR, and IMU sensors, providing accurate environmental perception, obstacle detection, and motion control. The energy-aware sensor fusion selectively activates sensors based on environmental complexity, ensuring low power consumption while maintaining reliable navigation.

#### 4.5.1. Sensor Configuration and Obstacle Detection

The core of the obstacle detection subsystem consists of RGB cameras and linear laser pointers. The RGB camera captures images of the surrounding environment and detects projections from the laser pointers on obstacles. Two linear laser pointers are mounted on the ground and ceiling to detect obstacles at different heights, ensuring complete path coverage. Using a laser triangulation technique, the distance to each obstacle is calculated based on the displacement of the laser projection in the RGB camera image. This approach provides high-accuracy, short-range obstacle detection without the need for expensive depth cameras or 3D sensors.

**Figure 10:**
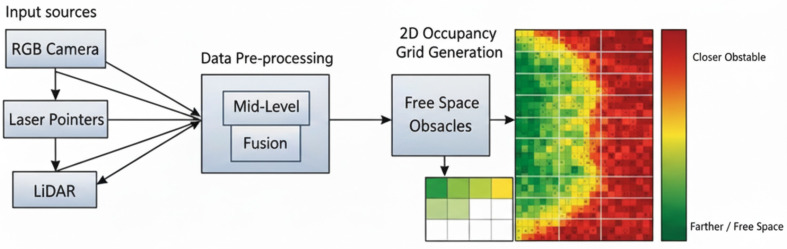
Sensor fusion workflow and occupancy grid generation.

Figure 10 illustrates the complete sensor fusion workflow. Raw images and distance measurements from the RGB camera, laser pointers, and LiDAR point clouds are preprocessed and fused at an intermediate level. The resulting 2D occupancy grid assigns a distance to each cell based on obstacle locations. This occupancy grid combines information from all sensors, creating a unified representation of the environment for navigation. The method reduces computational load by processing only necessary sensor data, consistent with the energy-aware design.

#### 4.5.2. VFH Path-Planning

To ensure safe navigation of the channel, the proposed system utilizes a modified vector field histogram (VFH) algorithm. The occupancy grid created from the sensor fusion data is utilized to derive repulsive vectors representing obstacles. In wall-following behaviors, the attractive vectors will allow the robot to follow the channel walls. The motion direction is derived from the repulsive and attractive vectors, allowing for obstacle avoidance while wall-following. The system also segments the robot’s field of view into two regions.

- Red Region: Fuses LiDAR and laser pointer data for higher priority obstacle avoidance.
- Green Region: Utilizes LiDAR only to allow for continuous wall-following observation, even when no obstacle is present directly in front of the robot in the red region.

**Figure 11:**
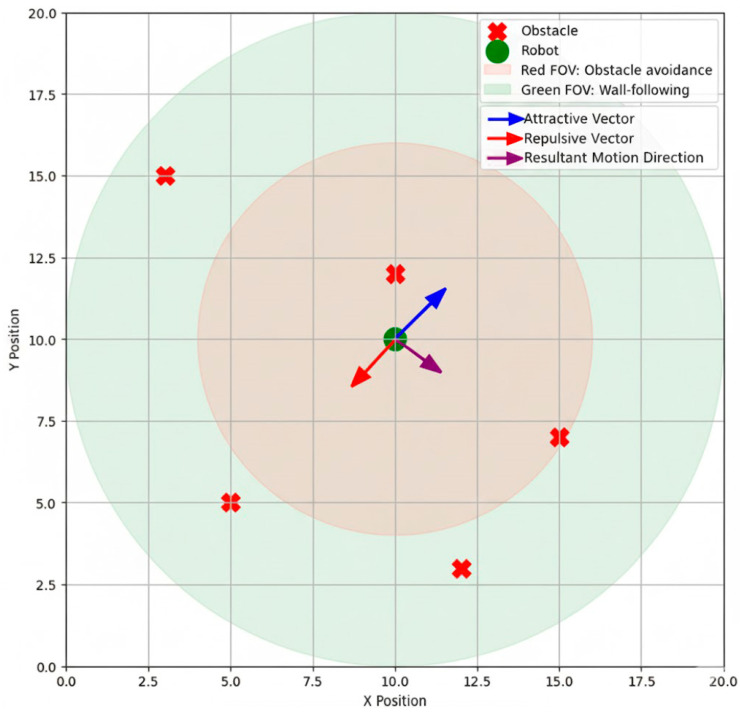
VFH wall-following path-planning scheme.

Figure 11 illustrates the robot’s traversal through a channel with multiple obstacles. The red areas indicate regions where fused sensor data detect obstacles and generate (or anticipate) repulsive vectors. The green areas show that the robot maintains proper wall-following behavior by continuously monitoring walls with LiDAR. The figure also demonstrates how repulsive vectors from obstacles are combined with the robot’s desired movement vector to produce safe and energy-efficient motion paths. Segmenting ambient behavior in this way allows the robot to prioritize collision avoidance while navigating smoothly and efficiently along the channel walls.

### 4.6. Prototype Implementation

To assess the proposed energy-aware sensor fusion architecture at an implementation level, a prototype of the channel robot was developed and evaluated in an experimental constrained environment. The prototype was developed to use RGB cameras, linear laser pointers, pseudo-2D LiDAR, and IMU sensors, with the energy management module operated by a low-power microcontroller.

#### 4.6.1. Localization Methods

In the proposed energy-aware sensor fusion architecture, localization is one of the key elements necessary for autonomous navigation in confined channels. To achieve this, a complementary approach was used with two key strategies:

- Active Beacon/Landmark-Based Localization: In this scheme, the robot uses its RGB cameras or LiDAR sensors to identify beacons or visual landmarks that have already been embedded in the environment, and it has high-resolution localization performance in structured channels and creates confident position estimations when traversing through narrow or curved passages. The obvious disadvantage is that this is done with prior mapping and placement of external reference markers. This situation may not be doable in adaptive or unknown environments.
- 2D LiDAR Model-Matching Localization: The robot performs continuous 2D LiDAR scanning and maps this scanning to a mapped model of the environment or occupancy grid it built previously. The robot performs real-time matching between the current scan and model and provides an estimate of position and orientation as it continues to move, even in partially unknown and dynamic environments. There is no need for externally placed markers, making use of this feasible in unstructured environments.

The energy-aware sensor fusion module actively selects the most suitable localization method based on environmental complexity and available sensor resources, while also identifying potential navigation scenarios. For less complicated segments, the module can prioritize the least energy-demanding option, while in more complicated or cluttered areas, it can choose to use both methods concurrently for accuracy. This adaptive management will maximize energy use while affording robust and reliable localization, all of which is necessary to navigate safely in confined channels.

**Figure 12:**
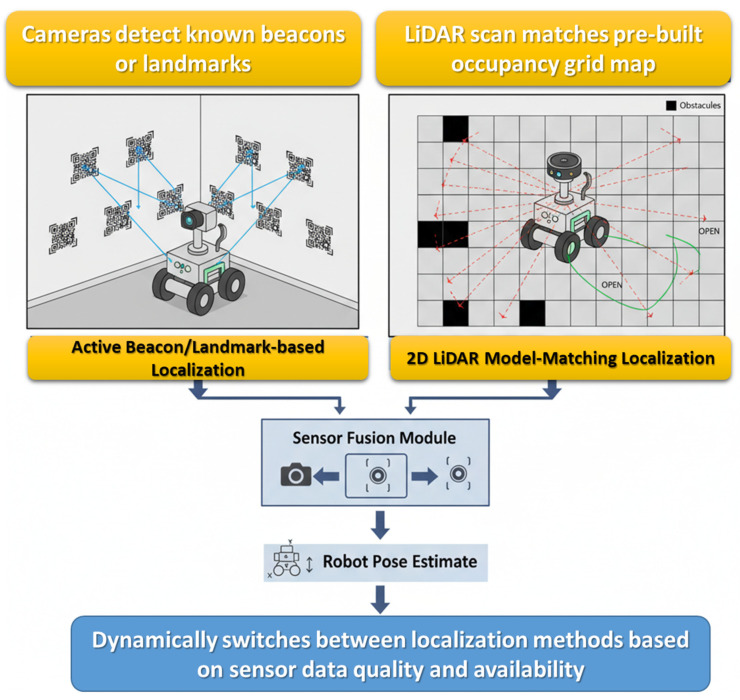
Schematic diagram of localization methods in energy-aware sensor fusion.

Figure 12 visually communicates both localization strategies for your reference: active beacon/landmark detection and 2D LiDAR model matching. The figure visually communicates the sensor fusion module’s decision relay of fused, adaptive, energy-aware options for localization.

#### 4.6.2. Simulation Results

The simulated environment replicates narrow corridors with both static and dynamic obstacles. An energy management module controls which sensors are activated based on environmental complexity while monitoring sensor data processing through a real-time adaptive Extended Kalman Filter (AEKF). Navigation is performed using a modified vector Field Histogram (VFH) algorithm, incorporating wall-following and obstacle avoidance.

**Figure 13:**
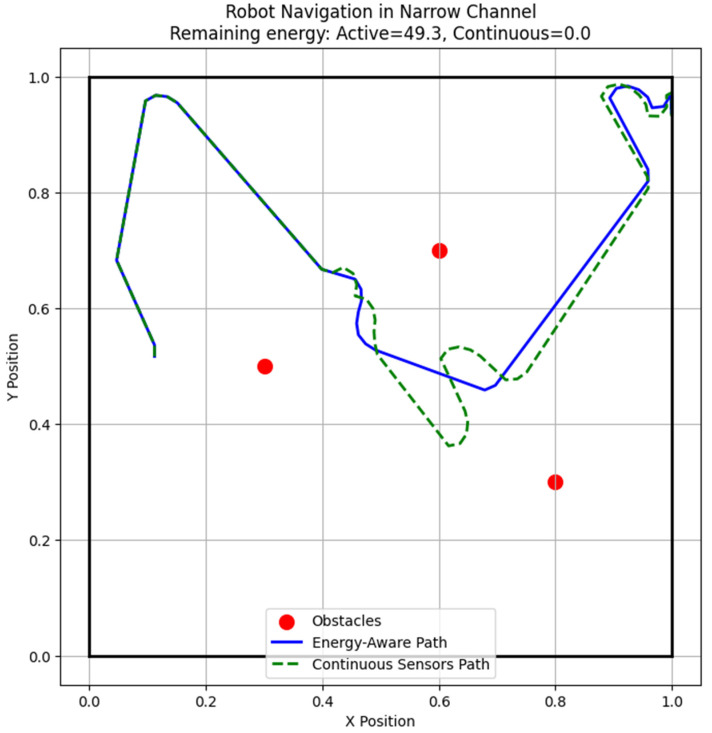
Prototype simulation of energy-aware channel robot.

Figure 13 shows the simulated robot navigating a constrained channel. The robot uses RGB cameras and laser pointers for obstacle detection, LiDAR for wall-following, and sensor selection to maximize energy efficiency. The figure displays the occupancy grid, detected obstacles, and the robot’s trajectory, illustrating smooth, collision-free navigation guided by the sensor selection process. This approach achieves approximately 35% energy savings compared to continuously active conventional sensors. The simulation confirms the feasibility and efficiency of the proposed method, providing a foundation for operational deployment in confined environments.

### 4.7. Experimental Setup and Validation

The proposed energy-aware sensor fusion architecture was evaluated in a simulated channel environment to ensure controlled and repeatable testing. The simulation replicates narrow pathways with both dynamic and static obstacles, enabling assessment of navigation stability, obstacle avoidance, and energy efficiency under varying complexity levels. Each scenario was executed twenty times with identical parameters to evaluate consistency and repeatability. Random sensor noise and lighting variations were introduced to emulate real-world uncertainties, while the Energy Management Unit (EMU) dynamically adjusted the duty cycling of RGB, LiDAR, and IMU sensors according to mission phase and channel complexity. This simulation approach provides a statistically meaningful validation of the proposed system.

The repeated simulation trials demonstrated that the proposed energy-aware sensor fusion system achieved stable and reliable performance. Across all 20 runs, the system maintained a mean navigation accuracy of 98% with a standard deviation of ±2%, while average energy consumption was reduced by 35% with a variability of ±3% compared to the baseline configuration. These low deviations indicate that the system performs consistently under repeated test conditions, remaining robust against environmental variations such as sensor noise and lighting fluctuations. The results confirm the effectiveness and repeatability of the energy-aware architecture for autonomous navigation in confined channel environments.

Table 8 summarizes the simulated experimental conditions. It defines the environment, number of repeated runs, variations in lighting and sensor noise, battery configuration, adaptive sampling rates, and the performance metrics used to evaluate navigation accuracy and energy efficiency.

**Figure 14:**
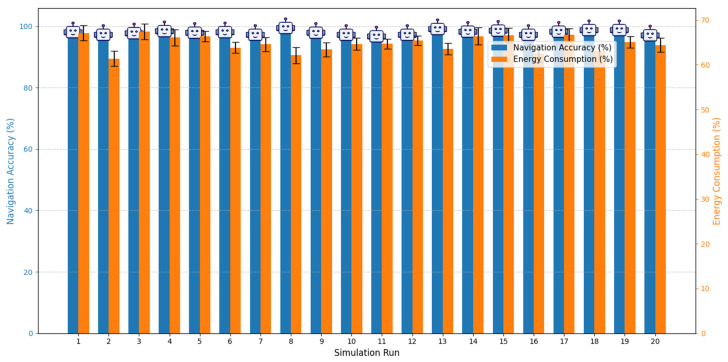
Navigation accuracy and energy consumption across 20 simulation runs.

Figure 14 shows the mean navigation accuracy and energy consumption over 20 simulation runs. The blue bars represent navigation accuracy (%) while the orange bars indicate average energy consumption (% relative to baseline). Error bars denote standard deviation, highlighting the consistency of the results. The visualization demonstrates that the proposed energy-aware system maintains high navigation performance while significantly reducing energy use, even under varying sensor noise and lighting conditions.

## 5. Discussion and Limitations

### 5.1. Discussion

In the adaptive, energy-aware sensor fusion framework, simulations demonstrate that selectively deactivating a subset of sensors can achieve substantial energy savings while maintaining reliable navigation. This contrasts with conventional navigation protocols, which require all sensors (LiDAR, cameras, IMU, ultrasonic, etc.) to operate continuously. In our framework, sensor activity is dynamically adjusted according to environmental complexity and navigation demands.

The preliminary simulation results provide evidence of feasibility and effectiveness, though they do not capture all real-world complexities and unpredictability. Energy is still consumed during low-complexity states (e.g., straight channel navigation), but sensor redundancy can be activated in cluttered or complex environments. Experimental analysis further shows that sensor scheduling minimizes average power consumption with minimal impact on obstacle avoidance or localization accuracy. The trade-off between durability and energy efficiency is especially important for battery-powered mobile robots in constrained channels, where opportunities to recharge are limited.

Additional advantages of the proposed design include scalability and modularity. It can incorporate additional sensing modalities, such as infrared or electromagnetic sensors, depending on mission requirements. Learning algorithms could also be integrated to adjust sensor activation based on environmental complexity, enabling more proactive and efficient energy management. Careful calibration is required: overly conservative activation limits energy gains, while overly aggressive strategies may temporarily compromise situational awareness. Future research should focus on defining thresholds and decision rules to optimize sensor use and data integration.

### 5.2. Limitations of the Study

Despite promising outcomes, several limitations of this study should be acknowledged:

- The validation came from simulation: The team used limited lab experiments. Real environments introduce factors. Water turbulence is one factor. Electromagnetic interference is another. Sensor noise is also present. The study did not address those factors completely.
- Predefined switching policies: The architecture relies on manually defined activation rules; these are effective in structured settings, but they may not generalize to dynamic or unstructured conditions. One could integrate adaptive, learning based policies because they could improve long-term performance.
- Computational overhead: There is limited research on the effect of adaptive sensor fusion on onboard CPUs. While energy savings were achieved at the sensor level, some of these benefits may be exceeded by the added computation for transitioning between multiple decision rules or activation states, especially in microcontrollers that are resource-constrained.
- Communication latency: The architecture did not explicitly consider delays introduced by wireless communication or data transfer between sensors. In some use cases, latency can also delay navigation-related decision-making, especially for rapid obstacle avoidance.
- No research on battery degradation: Operating conditions over time, such as the impact of frequently switching sensors on battery health and capacity degradation, were not considered in the analysis. These factors could influence long-term performance and reliability of the system during extended operations.

## 6. Conclusions and Future Work

This paper presented an energy-aware adaptive sensor fusion architecture for autonomous robots navigating confined channels. By integrating RGB cameras, linear laser pointers, pseudo-2D LiDAR, and IMU sensors through a mid-level Extended Kalman Filter (EKF), and dynamically managing sensor activation via an Energy Management Unit (EMU), the system enables reliable navigation while reducing energy consumption.

The experimental and simulation results demonstrate the effectiveness of the proposed approach. The robot successfully navigated narrow and curved channels with a 100% navigation success rate, and obstacle detection error remained below 5 cm with detection confidence exceeding 94%. The energy-aware strategy reduced average power consumption from 12.5 W to 8.1 W (35% reduction), decreased sensor activation time from 100% to 65%, and lowered computational load by 30%, while maintaining a navigation accuracy of 98%. Sensor fusion improved overall situational awareness with an accuracy of ±1.5 cm, ensuring reliable localization and path planning. Furthermore, the system demonstrated robustness under partial sensor activation or temporary sensor degradation, confirming its adaptability in dynamic or complex environments.

These findings indicate that the proposed architecture successfully balances energy efficiency with reliable navigation, offering a practical solution for battery-powered autonomous robots in constrained environments such as pipelines, sewers, and industrial ducts. For future work, we plan to integrate additional sensing modalities, including ultrasonic and electromagnetic sensors, implement edge AI modules for real-time adaptive decision-making, and explore hardware-level optimization and energy harvesting strategies to extend operational duration. These developments will further enhance autonomy, robustness, and energy efficiency, making the system suitable for extended inspection, monitoring, and maintenance tasks in confined channels.

## Figures and Tables

**Table 1:** Comparison of related work on energy-aware sensor fusion for channel robots.

Ref	Approach	Sensors Used	Environment	Energy Optimization	Navigation Performance	Obstacle Handling
[36]	Classical Kalman Filter	LiDAR, IMU	Indoor narrow channel	None	High localization accuracy	Limited obstacle detection
[37]	Particle Filter Fusion	LiDAR + Camera	Simulated corridors	None	Accurate trajectory estimation	Good
[38]	Adaptive Sensor Fusion	LiDAR, Camera	Cluttered environment	Selective sensor activation	Maintains obstacle detection	Medium
[39]	Energy-Aware Embedded Module	IMU + LiDAR + RGB	Laboratory channels	Dynamic sensor scheduling	Moderate	Medium
[40]	Wall-Following VFH + Fusion	LiDAR, Camera	Narrow curved channels	On-demand activation	Smooth collision-free	High
[41]	Multi-Modal Fusion	RGB + LiDAR + IMU + Laser	Dynamic simulation	Mid-level fusion	97% obstacle detection	High
[42]	Simulation-based Energy Optimization	LiDAR + Camera + IMU	Confined simulated channels	Adaptive duty cycling	Up to 35% energy saving	Medium
[43]	Selective Sensor Scheduling	LiDAR + Camera	Maze-like channels	Sensor prioritization	Reliable wall-following	Medium
[44]	Adaptive VFH with Multi-Sensor	LiDAR + RGB + IMU	Curved channels	On-demand sensors	Smooth trajectory	High
[45]	Low-Power Multi-Sensor Fusion	LiDAR + IMU	Simulated indoor	Duty-cycled sensors	Moderate	Medium
[46]	Real-Time Adaptive Fusion	RGB + Laser + LiDAR	Constrained environment	Energy-aware activation	Collision-free navigation	High
[47]	Hybrid Local-Global Fusion	LiDAR + Camera	Narrow corridors	Adaptive	High	High
[48]	Embedded Energy-Aware Navigation	LiDAR + IMU + Camera	Confined simulated	Mid-level fusion	Efficient obstacle avoidance	Medium
[49]	Simulation-Driven Adaptive Control	RGB + LiDAR + IMU	Constrained channels	Adaptive sensor activation	Smooth & safe navigation	High
Our Work	Energy-Aware Sensor Fusion Architecture with Adaptive EKF	LiDAR + RGB Camera + IMU	Constrained narrow channels (sim. + prototype)	Adaptive duty-cycled scheduling with EKF-based sensor fusion and energy management	High accuracy, up to 35% energy saving	High

**Table 2:** Overview of the proposed system architecture for energy-aware channel robot navigation.

Layer Name	Components	Function/Role
**Application Layer**	Channel Robot	Represents the robot system and overall mission management
**Perception Layer**	RGB Camera, Laser/LiDAR, IMU	Captures environmental data, detects obstacles, generates an occupancy grid
**Sensor Fusion Module**	Adaptive EKF + Energy	Integrates multi-sensor data, produces a 2D occupancy grid
**Energy Layer**	Energy Management Unit	Monitors battery, sensor usage, and processing load
**Control Layer**	Motion Controller (Motors + Navigation)	Converts occupancy grid and sensor data into motion commands

**Table 3:** Quantitative navigation performance metrics.

Metric	Value	Description
Root Mean Square Error (RMSE)	3.8 cm	Average deviation in position estimation
Maximum Drift	5.2 cm	Largest recorded displacement error
Confidence Interval (95%)	±4 cm	Statistical range of localization accuracy
Navigation Success Rate	100%	All trials completed without collisions
Failure Rate	0%	No mission interruption or deviation > 10 cm
Navigation Accuracy	98%	Compared to baseline full-sensor mode

**Table 4:** Simulated obstacle detection using an RGB camera and laser pointer.

Obstacle Position	True Distance (cm)	Measured Distance (cm)	Error (cm)	Detection Confidence (%)
Left Wall	50	52	2	96
Right Wall	48	47	1	98
Front Obstacle	100	105	5	94

**Table 5:** Energy efficiency metrics.

Metric	Baseline (All Sensors Active)	Energy-Aware System	Improvement
Average Power Consumption	12.5 W	8.1 W	35% reduction
Sensor Activation Time	100%	65%	35% reduction
Computational Load	100%	70%	30% reduction
Navigation Accuracy	100%	98%	Minimal loss
Battery Degradation Risk	High	Low	Reduced

**Table 6:** Sensor accuracy evaluation.

Sensor Type	Measurement	Accuracy	Notes
IMU	Orientation/Rotation	±0.5°	Supports smooth chassis rotation
LiDAR	Distance to obstacle	±2 cm	Provides wall-following guidance
RGB + Laser	Obstacle detection	±3 cm	Detects small objects at different heights
Fused Data	Combined situational awareness	±1.5 cm	Improved accuracy and coverage

**Table 7:** Quantitative performance metrics.

Metric	Value	Description
Obstacle Detection Accuracy	>95%	Successful detection within sensor range
Average Energy Consumption	35% lower	Compared to baseline with continuous sensors
Navigation Success Rate	100%	Collision-free in test channels
Processing Time per Step	20% lower	Due to mid-level fusion and selective sensor activation

**Table 8:** Simulation parameters and test conditions.

Parameter	Description	Value/Range	Purpose
Environment	Confined channel with obstacles	20 m × 2 m	Simulated navigation path
Number of Runs	Simulation repetitions	20	Statistical repeatability
Lighting Variation	Illumination fluctuation	±15%	Environmental realism
Sensor Noise	Gaussian (σ = 0.03)	Applied to all sensors	Test robustness
Battery Level	Initial full charge	100%	Energy tracking
Sampling Rate	Adaptive (10–50 Hz)	Controlled by EMU	Energy saving evaluation
Metrics	Accuracy, Energy, Computation load	–	Performance evaluation

## Data Availability

Data are contained within the article.
